# A New Wrist–Forearm Rehabilitation Protocol Integrating Human Biomechanics and SVM-Based Machine Learning for Muscle Fatigue Estimation

**DOI:** 10.3390/bioengineering10020219

**Published:** 2023-02-06

**Authors:** Yassine Bouteraa, Ismail Ben Abdallah, Khalil Boukthir

**Affiliations:** 1 Department of Computer Engineering, College of Computer Engineering and Sciences, Prince Sattam bin Abdulaziz University, Al-Kharj 11942, Saudi Arabia; 2 Control and Energy Management Laboratory, National School of Engineering of Sfax, Higher Institute of Biotechnology of Sfax, University of Sfax, Sfax 3038, Tunisia; 3 Research Laboratory in Intelligent Machines, National Engineering School of Sfax (ENIS), University of Sfax, Sfax 3038, Tunisia

**Keywords:** wrist–forearm biomechanics, robotic rehabilitation, human robot interaction, SVM classifier, machine learning

## Abstract

In this research, a new remote rehabilitation system was developed that integrates an IoT-based connected robot intended for wrist and forearm rehabilitation. In fact, the mathematical model of the wrist and forearm joints was developed and integrated into the main controller. The proposed new rehabilitation protocol consists of three main sessions: the first is dedicated to the extraction of the passive components of the dynamic model of wrist–forearm biomechanics while the active components are extracted in the second session. The third session consists of performing continuous exercises using the determined dynamic model of the forearm–wrist joints, taking into account the torque generated by muscle fatigue. The main objective of this protocol is to determine the state level of the affected wrist and above all to provide a dynamic model in which the torque generated by the robot and the torque supplied by the patient are combined, taking into account the constraints of fatigue. A Support Vector Machine (SVM) classifier is designed for the estimation of muscle fatigue based on the features extracted from the electromyography (EMG) signal acquired from the patient. The results show that the developed rehabilitation system allows a good progression of the joint’s range of motion as well as the resistive-active torques.

## 1. Introduction

Over the past few decades, robots have been essential to the rehabilitation process as they help stroke survivors restore motor function. Robots can expedite therapy, assist with patient mobility, and offer feedback. Numerous developments in robotics and information technology have helped automated rehabilitation systems [1,2,3]. In this context, we aim to develop a novel rehabilitation protocol based on human forearm–wrist biomechanics by utilizing the advancements gained in the mathematical modeling of human joints, Internet of Things (IoT)-based system design, and SVM-based decision-support systems.

Stroke is the main factor for serious long-term disability worldwide. The cost of rehabilitation and transportation, in addition to the lack of qualified staff and equipment, are all obstacles. A promising approach to lower costs, increase accessibility, and maintain patient autonomy is telerehabilitation (TR). TR enables healthcare professionals to deliver therapy remotely, potentially expanding access to underdeveloped areas. In [4], a recent review of control methods for upper limb telerehabilitation with robotic exoskeletons is presented. Moreover, several works present a new telerehabilitation platform with robotic devices for the at-home rehabilitation context [5,6,7]. In order to study the impact of telerehabilitation for stroke-related deficits, recent research reviews this subject to assess if TR is as effective as conventional in-person outpatient therapy in enhancing satisfaction and poststroke residual impairments such as motor function, speech, and disability [8].

Understanding the dynamics of forearm–wrist rotations is important for many fields, including biomechanics, rehabilitation, and motor neuroscience. Even though the underlying dynamics are typically complicated, human movement typically incorporates many degrees of freedom (DOF) coordinated smoothly and easily. It is crucial to understand these dynamics because they highlight the difficulties of the neuromuscular system in managing movement. Indeed, the torque necessary to overcome the passive mechanical resistance of wrist rotation is very usefully specified by the mathematical model of the wrist–forearm joint dynamics. The equations defining the motions of the wrist movement are based on many parts including inertial, damping, and stiffness [9,10]. These subject-related parameters can be measured experimentally to ascertain their value.

This active torque of the wrist joint is very sensitive to muscle fatigue. Physical exhaustion is, in fact, one of the most common symptoms of a variety of diseases, from multiple sclerosis and stroke to chronic insomnia and myo-skeletal injuries [11]. However, because tiredness is a subjective concept, there is still a great deal of untapped ground in terms of understanding, measuring, and forecasting fatigue occurrences. Each person experiences exhaustion in a unique way that fluctuates in its intensity and is influenced not only by their physiological condition but also by subjective aspects such as mood, which are exceedingly challenging to accurately identify [12]. Given that our failure to effectively record and forecast exhaustion could raise the risks of unintended injuries and muscular weariness, such occurrences might have detrimental effects on physical recovery. This fact is made much more crucial when considering autonomous rehabilitation systems and the requirement to create adaptive systems that are skill-appropriate for users. In that context, comprehending physical weariness has developed into a topic of intense scientific interest because of its significance for obtaining successful recovery [13]. Numerous articles were published over the past 20 years that suggested that modeling techniques and characteristics extract useful data from EMG [14,15,16,17,18,19,20,21,22]. In [14], a new concept for a lightweight exoskeleton rehabilitation robot controlled by surface electromyography was presented for at-home progressive resistance training. The lightweight exoskeleton’s construction was created using measurements of the arm’s ergonomic sizes and the kinematic model of the elbow joint. In [15], authors describe a hand exoskeleton controlled by electromyography (EMG) for fundamental motions in aided bilateral treatment when the user has to utilize both hands. Individuals with a right hand afflicted by an accident or cerebrovascular issues who need passive or aided rehabilitation are the intended users. On the other hand, an actuated glove that is compliant and has a control system to identify the user’s motion intent was presented [17]. This motion intent is determined by a machine learning algorithm based on muscle activity. Moreover, for the lower limb rehabilitation robot, scientists created a surface electromyography-based gain-tuned compliance control (EGCC) method [19]. Otherwise, to examine the techniques of EMG parameter identification and dynamic feature extraction, and to get the EMG characteristics and variation rules linked to human motion patterns, EMG signal analysis and feedback control were integrated into the virtual rehabilitation system [20]. In this study [20], a virtual reality training system for rehabilitation was developed, and a patient trial was used to validate the system’s efficacy. In similar research, the RobHand (Robot for Hand Rehabilitation) system, which consists of an exoskeleton that supports EMG-driven aided bilateral treatment utilizing a specially created low-cost EMG real-time embedded solution, was presented [21]. Indeed, RobHand was equipped with a threshold non-pattern recognition EMG-driven control that can recognize healthy hand motions and recreate them on the paretic hand’s exoskeleton. Although the control architectures of the mentioned previous works are based on the EMG signals, no muscle fatigue constraints were considered. To overcome this drawback, many recent works discuss the integration of muscle fatigue during interactions between subjects and assistive devices. In [23], the authors propose a self-adapting control method based on online fatigue detection in rhythmic arm movements. Moreover, the authors established a new assessment method of the finger rehabilitation effect, including three indicators of finger muscle strength, fatigue, and range of motion [24]. On the other hand, authors investigate a personalized upper limb rehabilitation system that is adaptive [25]. In order to adjust the forces utilized for rehabilitation (assistive and resistive) in real-time based on muscle fatigue, the system collects EMG data from the main muscles involved in movement [25]. It was established that various physiotherapists react differently to post-stroke fatigue. Some researchers [26] explored a survey on fatigue management in treatment sessions due to a lack of expertise in this field. It is necessary to stop a therapy session if a patient becomes fatigued in the middle of it. The most frequent effect is that a patient’s duration in a session is limited by muscle fatigue [27]. The main limitation of the cited works is that the rehabilitation session will be stopped when fatigue occurs.

In many articles on robotic rehabilitation, the human joint model has not been taken into account in the robot control architecture [28,29,30]. On the other hand, there are rare investigations that focus on the subject of fatigue during rehabilitation, whose authors try to interpret this fatigue by detecting muscle contractions [31,32]. In this article, on the one hand we take into account the human joint model and we integrate the torque produced by the patient as well as the torque generated by the robot in the dynamic model. On the other hand, the proposed architecture takes into account the muscular fatigue of the patient and its direct impact on the torque generated by the subject and consequently on the dynamic model of the system.

The rest of the paper is organized as follows: In the second section, the methodology is explained, including the design and modeling of robots, the modeling of wrist and forearm biomechanics, the control architecture, the pre-processing and extraction of EMG features, and the classification algorithm designed for estimating muscle fatigue. The third section presents a detailed analysis of the results.

## 2. Materials and Methods

### 2.1. Mechanical Design and Model

The capacity to help patients in regaining all three DOFs and the biggest Range of Motion (RoM) is crucial when building an exoskeleton or rehabilitation device for the wrist. According to the findings discussed in [33,34], the wrist RoM and finger motions were considerably impacted by a combination of forearm and wrist postures. People with hand motion impairment frequently have trouble manipulating their wrists. As a result, a lightweight, transportable forearm exoskeleton is sought, but most significantly, the rehabilitation tool must cover the area of the human hand with the largest workspace.

#### 2.1.1. Review of the Range of Motion of the Human Hand 

**Figure 1:**
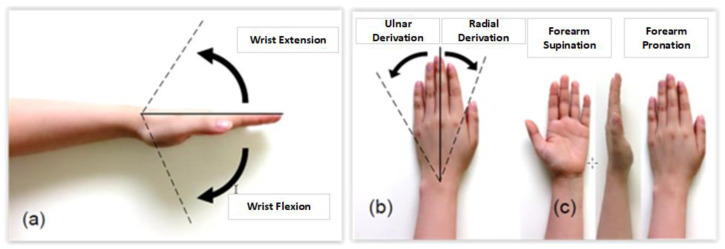
Movable RoM of the hand. (**a**) Wrist joint: extension and flexion, (**b**) Wrist joint: radial and ulnar deviation, (**c**) Forearm movement: supination and pronation.

The RoM of the human hand-on-wrist functioning, as described in the introduction and depicted in Figure 1, includes pitch, yaw, and roll. There are several different RoMs, according to recent investigations and records [35,36,37]. Therefore, the RoM of the human hand was studied and then contrasted with the RoMs utilized in other research in order to comprehend the ideal RoM of the wrist and hand. The RoM measurement technique as well as gender are variables that may impact the data. The RoM measurements revealed that women often exhibit more flexibility than males. As a result, if both men and women make up the population of the data, having different ratios of men and women can eventually alter the average value. The RoM utilized in this work was adjusted using the following values.: wrist extension (60°), wrist flexion (70°), ulnar deviation (30°), radial deviation (20°), forearm pronation (90°), and forearm supination (90°).

The following parameters were taken into account while determining the RoM to be employed for the proposed design: (1) The exoskeleton’s movable range should be less than the average value of the human hand’s movable range for safety. (2) The exoskeleton’s desired movable range may be wider than the exoskeleton’s average movable range. (3) Average data may not be the best suitable RoM to choose due to outlier data that could lower or raise the average. (4) The most often used RoM could be a better range than the typical range.

#### 2.1.2. Robot Design

From the design point of view, the goal is to design a high-precision stimulated rehabilitation system taking into account two main constraints; portability and lightness. Prior to making a final decision, a thorough analysis was done to determine the best-fit actuators. The motor torque necessary to elevate the complete upper limb should be the first parameter taken into account. Second, it is important to assure that the motions and position feedback are accurate. The torque produced by DC motors with gearboxes is adequate, but they lack positional precision. In order to identify the motor’s current location, rotary encoders must be fitted. Due to traffic problems, such a solution is not taken into consideration in this study. Stepper motors, which have a modest torque but average movement precision, are an additional choice. There are varieties with very high torque, but they are rather expensive. Precision feedback servomotors are used to provide quick and accurate integration and fixing, which primarily addresses space issues. However, the issue of restricted torque makes this method ineffective.

Our approach involves using two servomotors for each joint in order to address the torque issue while gaining the advantages of quick and accurate integration and repair. It is important to choose robot materials carefully since they affect a number of factors, including durability, and aesthetics. Other elements including weight (portability and lightness), simplicity of maintenance, potential extension, and cost should also be taken into account. The material and production are the major sources of cost. In fact, we decided to create a primary version of our robot to conduct some preliminary testing. The aluminum used for the final prototype was used to validate specific findings. The choice of aluminum is motivated by its lightness compared to iron and its cost compared to steel. Indeed, the system is intended for very fragile people so our robot must be light, giving the patient the possibility of using it standing or on a chair. In addition, the system must be able to support all of the elements to be integrated without any risk of a change in shape or characteristics.

**Figure 2:**
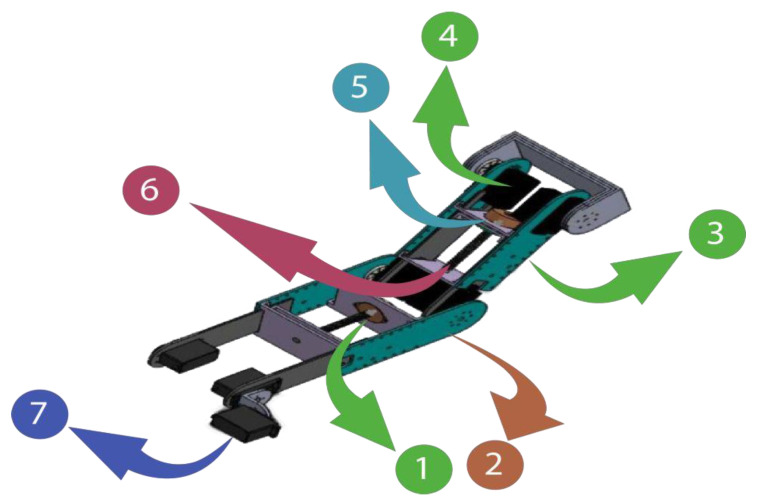
Three-dimensional design of the robot: (1) Inner plate; (2) Forearm outer plate; (3) Arm outer plate; (4) Giant scale servo motor-HS-805BB; (5) Nema stepper motor; (6) Screw-nut system; (7) Servo-motor HS-755HB.

The designed robot is distinguished by two detachable arm supports and can accommodate both the right and left arms. On the other side, there must be precise alignment between the patient’s joints and the system’s joints. The system’s extensibility solves this issue and ensures that it is suitable for all patients. The outer plates of the arm and forearm are regarded as the design’s focal points since they serve as the framework for the entire apparatus. Motors and bearings can be fixed using the holes on these plates. The bearing has an inner diameter of 4 mm, an exterior diameter of 9 mm, and a thickness of 4 mm. It can withstand forces of up to 70 kg (equivalent to 686.4 N). We employed a total of 48 bearings: 24 for the forearm and 24 for the arm. There are 12 bearings on each plate: 12 above and 12 below the inner plates. To provide friction-free translation of the inner plates, bearings are placed on the system’s outer plates. These mechanical adjustments are performed to position the robot in accordance with the patient’s arm measurements. The two outside plates of the forearm and arm are where the patient’s arm supports are fastened. The supports’ ability to move linearly enables the robot to be adjusted in accordance with the patient’s arm measurements. These brackets are moved by stepper motors and a screw-nut mechanism. In fact, we have incorporated two stepper motors for the screw-nut systems. Figure 2 depicts the mechanical layout of the suggested robot. Table 1 displays the features of the employed actuators.

#### 2.1.3. Robot Kinematics

**Figure 3:**
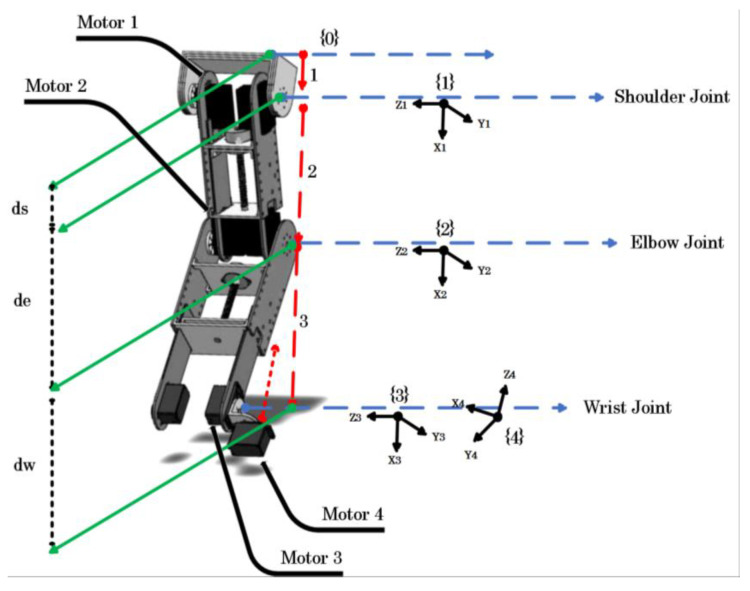
Robot kinematics.

The modeling of the exoskeleton was conducted based on the joints and movements of the human upper limb. In the model shown in Figure 3, joint {1} represents the shoulder joint which corresponds to the vertical extension/flexion of the shoulder joint. Joint {2} corresponds to the flexion/extension of the elbow joint. Joint {4} represents supination and pronation of the forearm and joint, respectively, and joint {3} corresponds to ulnar/radial deviation and flexion/extension of the wrist joint according to the four joint configurations, respectively. Indeed, supination and pronation of the forearm can be performed by actuating the motor {4} in the useful range [−85°; +85°]. In this case, the motor {3} is latched in a static position (usually at 0° to keep the forearm in a plane configuration). On the other hand, a fixed position of the motor {4] can configure the wrist joint for both flexion and extension movement (when the motor {4} is fixed at 0°) and the ulnar/radial deviation (when the motor {4} is fixed at 90°). The two derivations of the wrist are actuated by the motor 3.

The workspace of the exoskeleton is presented in Table 2. The kinematic analysis (Table 3) of the exoskeleton is based on the frames attached as shown in Figure 3.

### 2.2. Second-Order Mechanical Impedance Model of Wrist and Forearm Rotations

The axes of all three DOF intersected at the same location when the wrist and forearm joints were represented as a universal joint. Although it is thought that the Radial–Ulnar Derivation RUD axis is slightly distal to the Flexion–Extension FE axis, this distance is insignificant and was demonstrated to have no impact on wrist dynamics [38]. The following equations of motion relate the torque in each DOF to the resultant movement and account for inertial, damping, stiffness, and gravitational influences for each DOF:

$$\left[\begin{array}{c} M_{\alpha }\\ M_{\beta }\\ M_{\gamma } \end{array}\right]=\left[\begin{array}{ccc} A & B & C\\ B & D & E\\ C & E & F \end{array}\right]\left[\begin{array}{c} \ddot{\alpha }\\ \ddot{\beta }\\ \ddot{\gamma } \end{array}\right]+\left[\begin{array}{c} G\\ H\\ I \end{array}\right]+\left[\begin{array}{ccc} B_{\alpha \alpha } & B_{\alpha \beta } & B_{\alpha \gamma }\\ B_{\beta \alpha } & B_{\beta \beta } & B_{\beta \gamma }\\ B_{\gamma \alpha } & B_{\gamma \beta } & B_{\gamma \gamma } \end{array}\right]\left[\begin{array}{c} \dot{\alpha }\\ \dot{\beta }\\ \dot{\gamma } \end{array}\right]+\left[\begin{array}{ccc} K_{\alpha \alpha } & K_{\alpha \beta } & K_{\alpha \gamma }\\ K_{\beta \alpha } & K_{\beta \beta } & K_{\beta \gamma }\\ K_{\gamma \alpha } & K_{\gamma \beta } & K_{\gamma \gamma } \end{array}\right]\left[\begin{array}{c} \alpha \\ \beta \\ \gamma \end{array}\right]\;+glm_{h}\left[\begin{array}{c} \sin\alpha \sin\gamma -\cos\alpha \cos\gamma \sin\beta \\ -\cos\beta \cos\gamma \sin\alpha \\ \sin\alpha \sin\beta \sin\gamma -\cos\alpha \cos\gamma \end{array}\right]$$

Active torques are shown on the left side of Equation (1) (due to muscle contraction), which, given that brain activity impacts the stiffness and damping of muscles, may rely on displacement and its derivatives. The passive inertial, damping, stiffness, and gravitational influences that the active torques must overcome in order to cause movement are included on the right-hand side of equation (1). The matrix containing elements A through F is the inertia matrix, while G, H, and I contain the centripetal and Coriolis terms (Table 4). Passive damping and stiffness (i.e., in the absence of muscle contraction) are represented by the matrices containing damping coefficients (e.g., Bαα) and stiffness coefficients (e.g., Kαα), respectively. We made off-diagonal stiffness terms equal to each other since it has been demonstrated that the passive stiffness of the wrist and forearm is symmetric [39]. Parameters g, l, and m represent the gravitational acceleration, distance from the wrist joint to the center mass of the hand, and the mass of the hand, respectively. Model parameters are defined as follows:

Inertia. Equations for published anthropometric regression [11] were utilized to calculate the segment length measurements from each subject’s hand and forearm, as well as the mass and center of mass of the hand. These equations rely on the symmetry of the hand and forearm’s body-fixed inertia matrices (i.e., negligible products of inertia).

Stiffness. Each participant had their passive stiffness of paired wrist and forearm rotations quantified [39]. A rehabilitation robot specifically manipulated each subject’s wrist and forearm in a quasi-static way while measuring the displacement and the torque needed to achieve that displacement. The 3-DOF stiffness matrix was then computed using multivariable linear regression using the torque and displacement data.

Damping. Although the passive damping connected to linked wrist and forearm rotations is unclear, damping in flexion–extension was measured to be 0.02–0.03 Nms/rad [40]. There is an approximately proportionate relationship between the stiffness and damping ellipses associated with shoulder and elbow motions according to several studies [41]. As a result of this, it was assumed that the damping of the wrist and forearm was proportional to their stiffness (the constant of proportionality was selected so that the damping in FE would be 0.03 Nms/rad).

Dominant impedance effect. For each movement, we determined the average magnitude of the torque vector (in all three degrees of freedom combined) needed to overcome each of these impedance effects, as follows:

$$M_{j}=\frac{1}{T}\int_{0}^{T}||{\vec{M}}_{\alpha j}+{\vec{M}}_{\beta j}+{\vec{M}}_{\gamma j}||\;dt=\frac{1}{T}\int_{0}^{T}\sqrt{M^{2}{}_{\alpha j}+M^{2}{}_{\beta j}+M^{2}{}_{\gamma j}+2M_{\alpha j}M_{\gamma j}\sin\beta }dt$$

where *j* represents the impedance element or gravity, and *T* is the duration of the movement.

Interaction between degrees of freedom. The DOF in a multi-DOF system are often coupled, which means that the torque in one DOF relies on movement not only in that DOF but also in the other DOF. In other words, the entire torque in a DOF may be split into an interaction torque and a primary torque. We calculated the ratio of the average magnitude of the interaction torque vector to the average magnitude of the main torque vector for each movement to determine the degree of coupling between DOFs:

$$R=\frac{\frac{1}{T}\int_{0}^{T}\left|\left|\;{\vec{M}}_{\alpha ,IT}+{\vec{M}}_{\beta ,IT}+{\vec{M}}_{\gamma ,IT}\right|\right|\;dt}{\frac{1}{T}\int_{0}^{T}\left|\left|\;{\vec{M}}_{\alpha ,MT}+{\vec{M}}_{\beta ,MT}+{\vec{M}}_{\gamma ,MT}\right|\right|\;dt}$$

where *IT* and *MT* stand for interaction torque and main torque, respectively. This definition states that a bigger value of *R* denotes a stronger link between DOFs. Only the diagonal components of each matrix were used to produce the main torques from Equation (1), whereas only the off-diagonal elements were used to obtain the interaction torques. Since it is impossible to separate the gravitational torque into its primary and interaction torques, gravitational effects were left out of this section of the analysis.

We also computed this ratio separately for inertia, damping, and stiffness:

$$R_{j}=\frac{\frac{1}{T}\int_{0}^{T}\left|\left|\;{\vec{M}}_{\alpha ,ITj}+{\vec{M}}_{\beta ,ITj}+{\vec{M}}_{\gamma ,ITj}\right|\right|\;dt}{\frac{1}{T}\int_{0}^{T}\left|\left|\;{\vec{M}}_{\alpha ,MTj}+{\vec{M}}_{\beta ,MTj}+{\vec{M}}_{\gamma ,MTj}\right|\right|\;dt}$$

where *j* represents the impedance element.

### 2.3. Control Architecture Design

**Figure 4:**
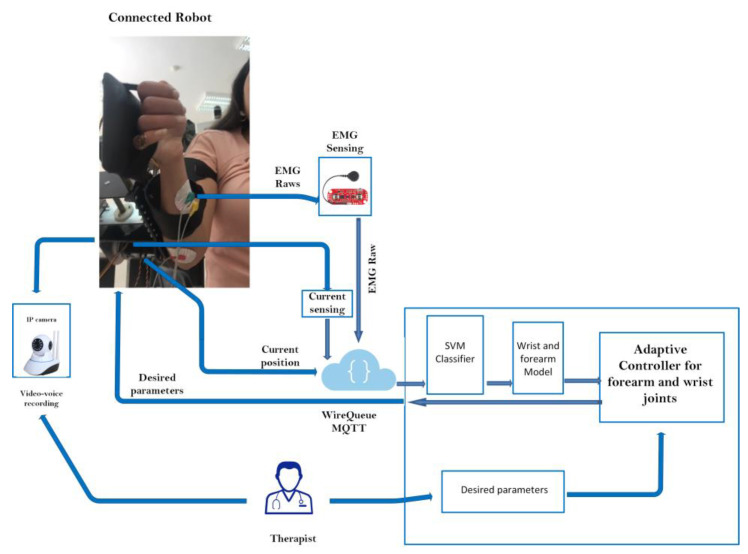
Control architecture overview.

**Figure 5:**
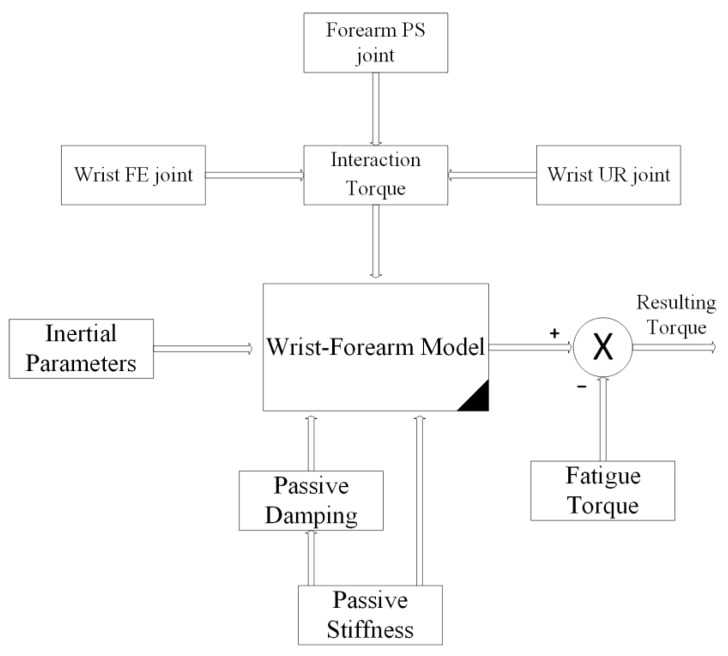
Wrist model and resulting torque.

**Figure 6:**
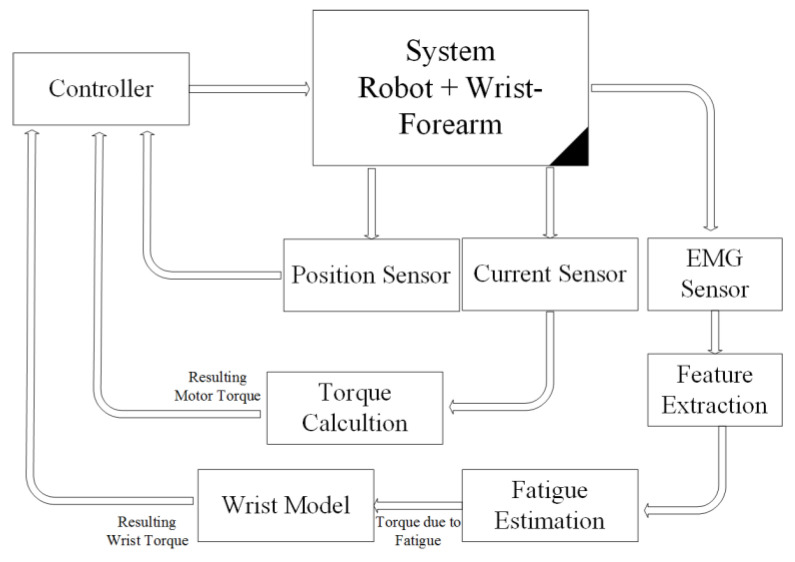
Loaded robot control architecture.

The primary architecture includes various subsystems, as seen in Figure 4. The physiotherapist initiates the process by entering the required settings into the LabVIEW-based human–machine interface (HMI). This HMI includes a sophisticated controller built using the wrist–forearm concept in use. The wrist model is continuously updated, as seen in Figure 5, in order to calculate the torque generated by the wrist–forearm joint. As indicated in Figure 6, the resultant torque may be computed by deducting the model-based output torque from the anticipated torque owing to fatigue. The muscle fatigue estimation block uses an SVM classifier to determine how tired the muscles are based on the attributes that were derived from the obtained EMG raw. On the other hand, the wrist’s current joint position is obtained via the associated encoder of the servo motor, and the motor torque is computed based on the implemented current sensor. The main controller, as shown in Figure 6, analyzes the inputs, including the current wrist joint position, the resultant wrist torque, and the produced motor torque, and sends the new required parameters to the robot. A real-time CompactRIO Single-Board Controller sbRIO-9627 is in charge of controlling the robot and assuring motor control; the real-time acquisition of the motor current from the current sensor to calculate the generated torque of the motor; and the real-time capture and processing of the EMG data. The sbRIO9627 is an embedded controller that integrates an I/O port, a user-configurable Field-Programmable Gate Array (FPGA), and a real-time central processing unit (CPU) running NI Linux Real-Time on a single printed circuit board (PCB).

#### 2.3.1. EMG Acquisition, Pre-Processing, and Feature Extraction

AgCl electrodes perform signal acquisition, and skin-electrode contact is ensured by following the guidelines of the surface EMG (sEMG) standards for the noninvasive evaluation of muscles (SENIAM) [42]. The volunteer’s skin was shaved, and an alcohol swab was used to clean the skin to enhance the signal acquisition further. The electrode position was according to the SENIAM recommendations; moreover, guidelines stipulate the distance between electrodes. The sEMG information was acquired from two forearm muscles: Flexor Carpi Ulnaris (FCU) and brachioradialis.

The acquisition system of the EMG data is ensured by the Myoware EMG sensor using the pre-gelled surface electrodes. This sensor can measure muscle activity by generating two outputs: the first is the raw EMG and the second is the rectified EMG using analog signal conditioning. In this work, we use the raw EMG as the input of the acquisition system using the real-time National Instruments board sbRIO-9637.

The amplifier gain of the acquired signals is set to 1000. The sampling frequency of the acquisition system is configured at 100 KHz with 10,000 samples/second. In addition, a second-order Butterworth-type band-pass infinite impulse response (IIR) filter is implemented with a set of 10–2000 HzA. With a reference touching the olecranon and the wrist joint, the pre-gelled surface electrodes were positionted on both sides of the associated muscles in order to provide two channels of EMG signals. In order to optimize the quality of the received signal, we design a first-order Butterworth-type notch IIR filter of 50 Hz to remove the power line noise in the LabVIEW block diagram. This filter is designed based on the digital filter design module for LabVIEW.

Based on the observation that muscle fatigue changes occur rather slowly [11], empirical window sizes were employed to divide the EMG data into short- and mid-term windows. Non-overlapping short-term windows with a length of 0.25 sec were extracted and overlapping mid-term windows with a length of 2 sec and a window step of 1 sec were extracted. The procedure of feature extraction involves removing the pertinent information from the EMG data and disregarding the rest. To effectively extract the data given by the EMG signal, signal processing and pattern matching approaches, such as the extraction of features and pattern classification, are developed. It is customary to explore the frequency or spectral domain features to investigate muscle fatigue. Power spectral density (PSD) analysis becomes crucial in the frequency domain. The SVM classifier in this section takes two time–frequency–domain feature extraction methods as inputs: the mean frequency (MNF) and the mean power (MNP).

The term “Mean Frequency” (MNF) refers to the average frequency that is determined by multiplying the total intensity of the spectrum by the frequency and the product of the EMG power spectrum [43]. The following formula may be used to compute it:

$$MNF=\sum_{j=1}^{M}f_{j}P_{j}\;/\;\sum_{j=1}^{M}P_{j}\;$$

where $f_{j}$ is the frequency of the spectrum at frequency bin *j*, $P_{j}$ is the EMG power spectrum at frequency bin *j*, and *M* is the length of the frequency bin.

The EMG power spectrum’s mean power (*MNP*) is the average power. The formula for the computation is

$$MNP=\sum_{j=1}^{M}P_{j}\;/\;M$$

For real-time data acquisition and processing, a LabVIEW interface is implemented. Indeed, the real-time acquisition is controlled by the interface providing a good tool for data monitoring and processing based on the official National Instrument libraries. Indeed, there are a few modules and packages to be installed to ensure the real-time performance of the acquisition system including the NI CompactRIO driver software, the LabVIEW FPGA Module, and the LabVIEW Real-Time Module. On the other hand, the feature extraction approaches were implemented by using the LabVIEW advanced signal processing toolkit as well as the biomedical toolkit. Notably, all processing is also ensured by the real-time NI board and the executed LabVIEW code is compiled and converted to VHSIC Hardware Description Language (VHDL) code via FPGA synthesizer and uploaded to the Ethernet-connected NI board.

#### 2.3.2. LabVIEW-Based SVM Classifier for Muscle Fatigue Estimation

**Figure 7:**
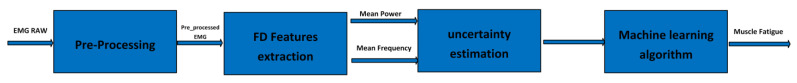
Muscle fatigue estimation approach.

One of the most prevalent signs of a wide range of illnesses, from multiple sclerosis and stroke to persistent insomnia and musculoskeletal injuries, is physical tiredness. However, because tiredness is a subjective concept, there is still a great deal of untapped ground in terms of understanding, measuring, and forecasting fatigue occurrences. Each person experiences exhaustion in a unique way that fluctuates in severity and is influenced not just by one’s physiological condition but also by subjective aspects such as mood, which are exceedingly challenging to accurately identify. Given that our failure to effectively record and forecast exhaustion could raise the risks of unintended injuries and muscular weariness, such occurrences might have detrimental effects on physical recovery. This fact is made much more crucial when considering autonomous rehabilitation systems and the requirement to create adaptive systems that are skill-appropriate for users. In this context, an issue that has drawn a lot of scientific attention is the awareness of physical weariness because of how crucial it is to successful recovery. The wrist torque is mostly reliant on the wrist model, as was previously described. However, the torque generated is extremely susceptible to muscular exhaustion. In order to calculate the actual resultant torque, the torque caused by muscle tiredness will be removed from the estimated torque based on the model. The fatigue torque must be directly calculated experimentally from natural muscle surfaces in order to ensure a trustworthy wrist torque computation. An SVM classifier is created and put into use utilizing LabVIEW software to accomplish this purpose. In fact, the SVM classifier uses two inputs derived from the feature extraction technique to determine the degree of muscle weariness. The muscular fatigue evaluation method is shown in Figure 7. The implementation of the SVM classifier is ensured by The LabVIEW Analytics and Machine Learning Toolkit. Indeed, The LabVIEW Analytics and Machine Learning Toolkit is a software add-on for LabVIEW that provides training machine learning models. We can use these models to discover patterns in large amounts of data with anomaly detection and classification and clustering algorithms. Additionally, these models can recognize patterns in new data on NI Linux Real-Time and Windows targets.

Support Vector Machines SVMs are supervised machine learning techniques that are widely utilized in density estimation, regression, and classification. One of the most reliable and accurate machine learning algorithms is said to be this one. A discriminating hyperplane is used by the SVM classifier to distinguish between the individuals in two groups. The input vectors are nonlinearly mapped to a very large feature space. The gap or separation between the closest training sets of two different classes is maximized by an ideal hyperplane. A quadratic optimization issue exists here. Depending on how easily training data points can be separated, the decision boundary of SVMs may be linear or nonlinear. The majority of the retrieved characteristics in real-world applications cannot be separated linearly. By utilizing the kernel method, a nonlinear decision boundary or hyperplane may be created with a minimal increase in computing complexity [43]. The kernels, including polynomial and radial basis functions, are taken into consideration in this study.

**Figure 8:**
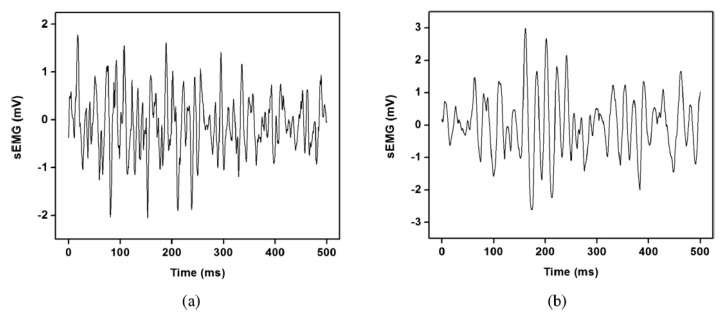
Representative (**a**) nonfatigue and (**b**) fatigue segment of sEMG signals.

Figure 8 displays the sample sEMG signal segments for muscular nonfatigue and fatigue states. The firing rate, motor unit recruitment patterns, muscle fiber conduction velocity, various muscle fiber types, and volume conductors are only a few of the physiological factors that affect the amplitude and frequency changes of the recorded signals.

In our study, 57 healthy participants are recruited to generate the dataset that will be used for SVM training the resulting model of muscle fatigue detection. Participants (totally active) are asked to use the rehabilitation robot (totally resistive) and the related Time-Frequency Domains TFDs features of the EMG signals were recorded during exercises. An uncertainty algorithm is used to lessen the uncertainty of the feature set. This algorithm chooses the salient characteristics that distinguish between muscular fatigue and nonfatigue states.

**Figure 9:**
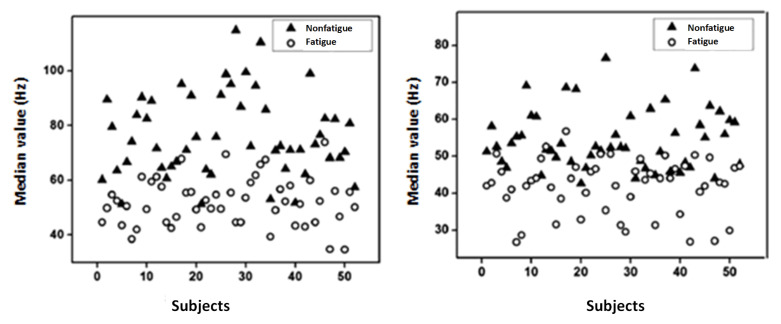
Median value extracted from (**a**) MNF and (**b**) MNP.

**Figure 10:**
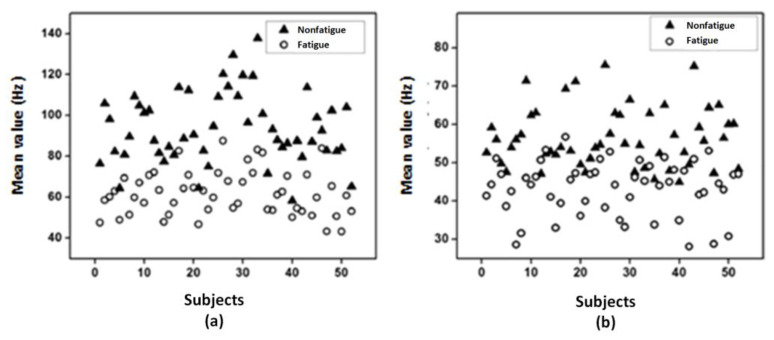
Mean value extracted from (**a**) MNF and (**b**) MNP.

Figure 9 and Figure 10 show the median frequency and mean frequency, respectively, computed from the considered feature extractions (MNF and MNP) for each subject. It is found that both the median value and mean value are higher in nonfatigue conditions and these results are in agreement with the literature [42,44]. The findings suggest that the suggested time–frequency method, including the MNF and the MNP, might be used in the study of muscle fatigue.

Table 5 displays the results of the TFDs using the two characteristics related to the SVM classifier. It is discovered that the chosen classifier achieves an accuracy of above 90%.

#### 2.3.3. Defined Rehabilitation Process

According to the patient’s and the robot’s existing conditions, the proposed rehabilitation procedure consists of three basic stages. The calculation of the subject-related constant parameters is the first stage in this process. Axis offset, inertial parameters, passive stiffness, and passive damping are all involved. The inertial parameters are measured directly from individuals, and axis offset is established as a constant. From the first exercise, when the force and position were collected from the completely passive patient, passive stiffness and damping will be assessed. The second phase is figuring out the wrist model’s remaining elements, including the interaction torque. The subject must make the proper motions in order to generate the torque required to drive the robot in the intended direction. In this instance, the robot uses a resistive torque to determine the torque the patient created. This stage, though, only applies to patients who are making moderate to rapid progress in their recovery. In fact, participants who are unable to deliver a minimal legible torque begin their activities passively, and their wrist model is entirely passive. However, because of muscle exhaustion, produced torque is a time-variant characteristic. As a result, it is necessary to do fatigue assessment using trustworthy natural interfaces like EMG signals. Total torque in upcoming workouts is the result of adding the torque generated by the robot and any torque supplied by the patient while taking into account fatigue limits. The evaluation of the rehabilitation state level and the extraction of the key elements of the wrist dynamic model are the two major objectives of wrist modeling.

#### 2.3.4. Tele-Rehabilitation Architecture

**Figure 11:**
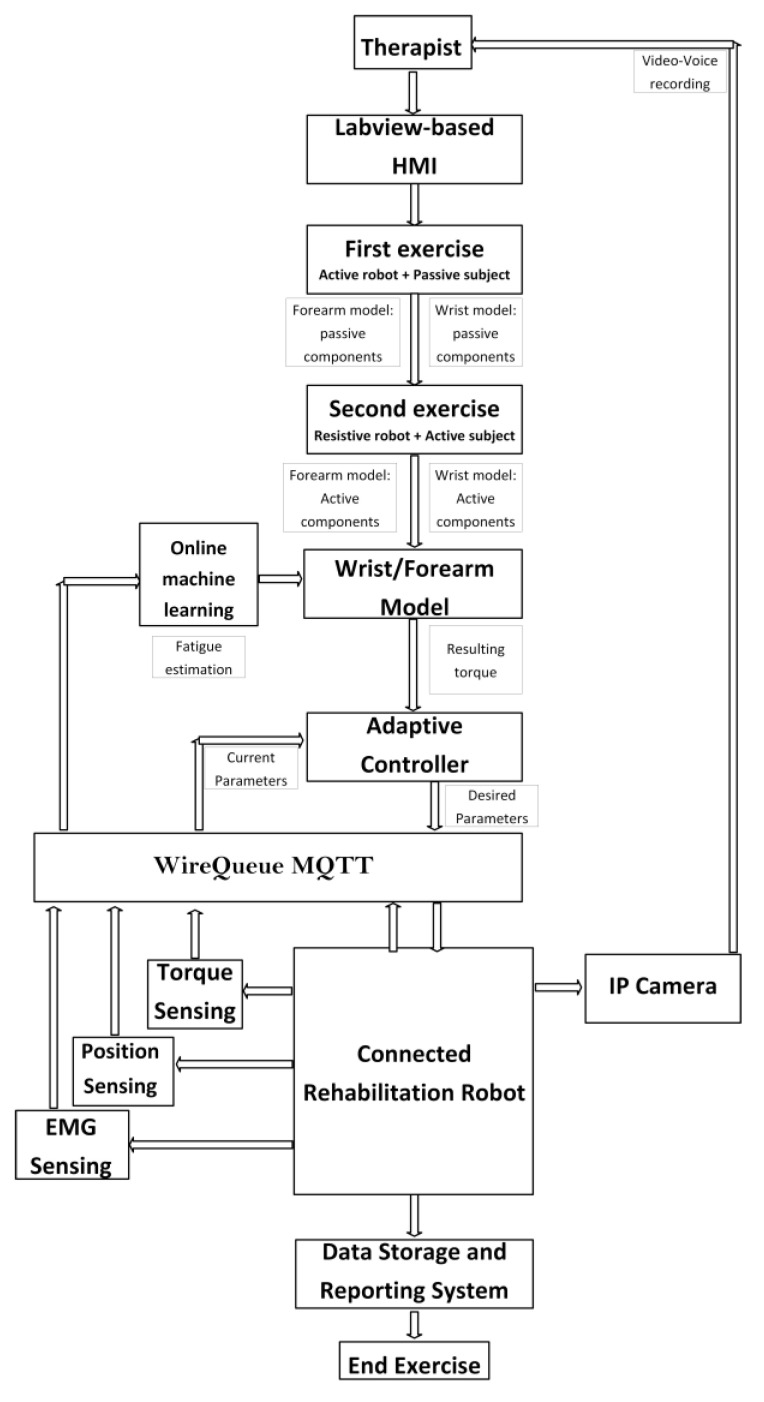
Operating system flowchart.

The physiotherapist enters the required settings to begin the control operation. After the necessary processing, the required parameters are sent to the sophisticated controller block implemented in the LabVIEW-based HMI. Through the Message Queuing Telemetry Transport (MQTT) protocol, the wrist robot gets the calculated parameters, including the required torque and desired position. Prior to returning the data to the control station through the MQTT protocol, the sb-RIO board processes the data from the current sensor, EMG sensor, and encoder. The estimated muscle tiredness is then calculated utilizing extraction techniques using the recovered frequential properties from the collected EMG raw. The SVM classifier, which determines the patient’s level of weariness, receives the EMG characteristics. Position feedback is used by the control motion block to determine the best option. The database keeps track of the options and the outcomes. Figure 11 depicts the operational flowchart of the system.

**Figure 12:**
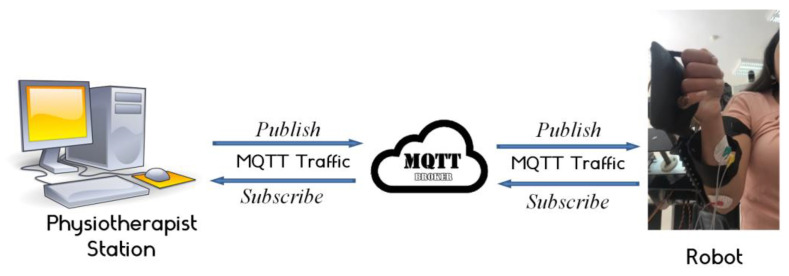
Iot platform.

The most crucial aspect of the MQTT protocol that sets it apart from others is its real-time requirement, which eliminates latency problems. Network consumption must be kept to a minimum to prevent interruptions brought on by the rise in demand for data exchange. By reducing the size of data packets, the MQTT protocols enable the network to be used more effectively and prevent network outages brought on by high data demand. The MQTT central server serves as a message dispatcher (also known as a broker). IoT devices may subscribe to or publish messages using this service. A client executes a “publish” action when it wishes to send data to the server. A client’s request to get data from the broker is known as a “subscribe” action. The devices subscribe to and publish topics. Controlling data flow between multiple devices is the broker’s responsibility. In the planned architecture, the physiotherapist’s role is to act as a broker. In this case, the subject to publish about is the computed torque. On the other side, the wrist robot has subscribed to this topic so that it may be informed of any developments. The physiotherapist’s station also requires input for the EMG raw, encoder position feedback, and current sensor feedback. Figure 12 depicts the actual architecture that was used.

## 3. Results and Discussions

**Figure 13:**
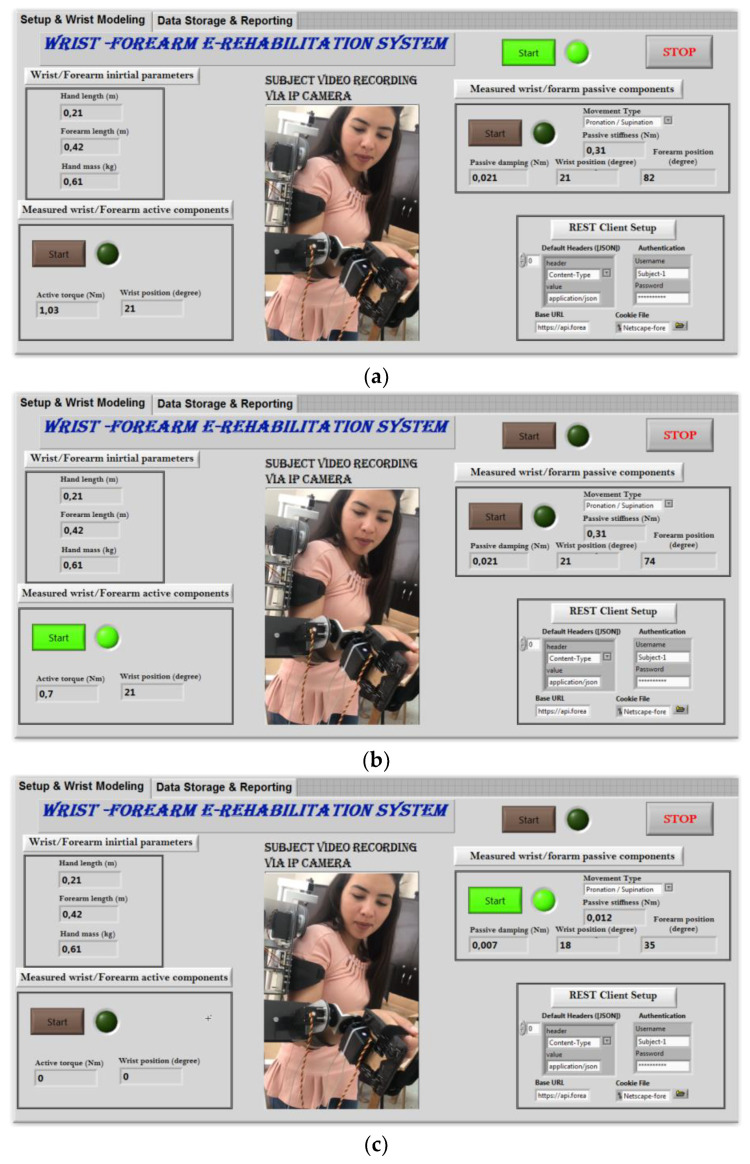
Setup sequences: (**a**) measuring passive wrist components; (**b**) measuring wrist active components; (**c**) continuous exercises.

The developed robot is a great option for at-home rehabilitation since it is portable, useful, and easy to operate. The patient finds it easier because they are not involved in the device’s functioning, and it is controlled remotely by a physiotherapist. In practice, following a stroke, the patient is generally sedentary and unable to do anything. This newly developed gadget offers a straightforward HMI with two distinct control screens. Figure 13 depicts the first, which is used as a configuration and supervision tool. At this point, the interface provides an authentication service. This service improves the networked system’s security. As seen in Figure 13, the system delivers real-time streaming. An IP camera that is integrated into the control architecture is used to provide movies to the router. On the physiotherapist’s PC which is connected to the router, a live video of the patient is seen during the rehabilitation process. The physiotherapist must first enter the patient’s wrist characteristics, including the patient’s hand length, forearm length, and hand mass (Figure 13). Notably, these parameters will be imported later in the subsequent exercises and are automatically stored in the relevant database. When something changes, the physiotherapist can alter these settings at any moment. The following stage involves starting the first workout and monitoring the wrist’s passive components (Figure 13a). The physiotherapist should use flexion/extension exercises or ulnar/radial derivations as the workout type. To identify the passive components of the totally passive patient at this phase, the robot may go through a few rounds. The convergence of the calculated data is mostly influenced by the number of iterations. The convergence of these data will provide visual information to the user. The linked database stores the passive components’ final values for evaluation. The wrist joint’s active components are next measured once the measurement of the passive components is complete. Both the active torque and the accomplished range of motion are noted during this phase. In this instance, the patient is fully engaged, and the rehabilitation robot applies a resistive torque. The patient is instructed to make the necessary movement while overcoming the robot’s resistive torque. To assess the degree of rehabilitation, both the maximal active torque generated and the acquired range of motion will be recorded. During the ongoing rehabilitation activities, the control system makes use of both passive and active components.

**Figure 14:**
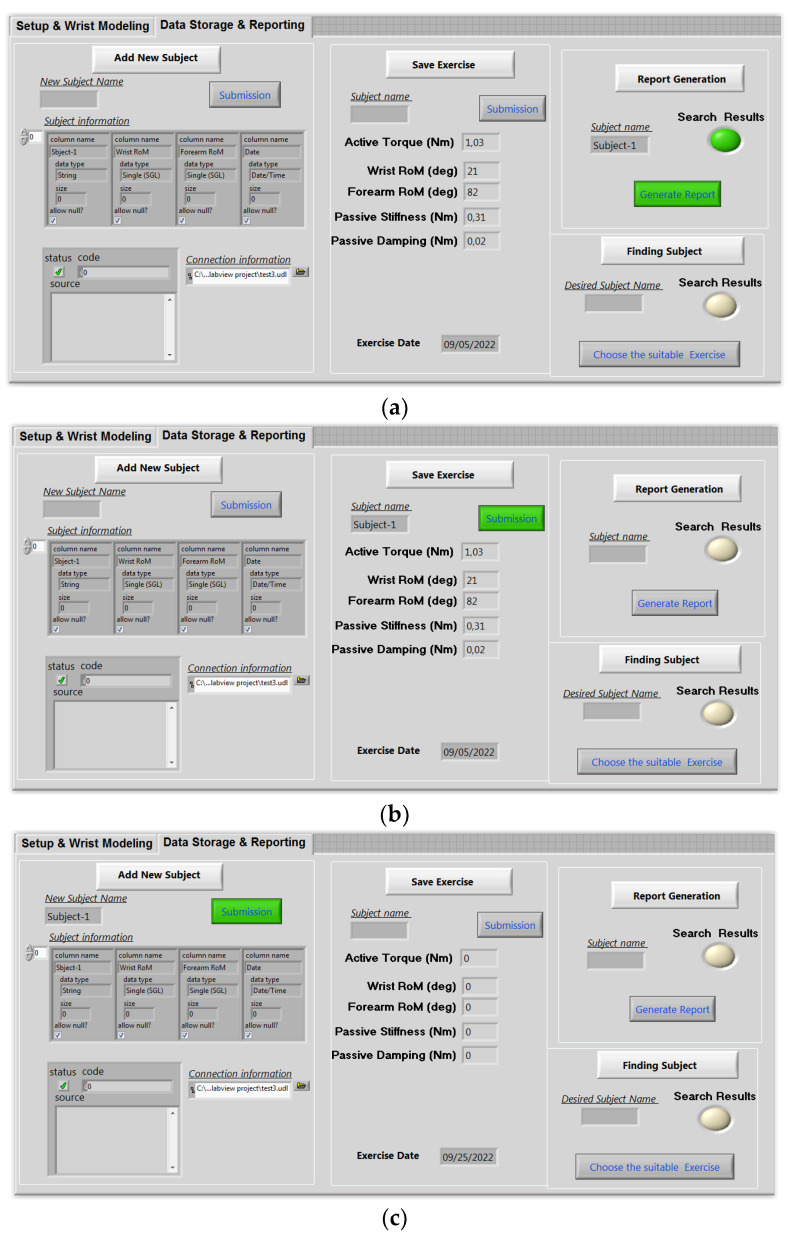
Data base interface: (**a**) add new subject; (**b**) save exercise; (**c**) generate report.

The data storage and reporting interface are displayed on the second screen of the user interface. The physiotherapist may use this screen’s capabilities to add a new subject (Figure 14a), save a new exercise (Figure 14b), and create the corresponding report (Figure 14c). In truth, electronic health records may provide both patients and physicians with several benefits. Remote access is possible for groups of patient records. As a consequence, it is easier to transfer a patient between rehabilitation facilities and monitor the development of his medical file. In this situation, the software developed, as shown in Figure 14, enables the recording of patient follow-up reports, workouts, and personal data. One week after the cast is removed, three individuals, each of whom has a fractured wrist, are tested.

Experiments were performed on three stroked subjects, all with wrist disabilities. Table 6 presents the initial states/obtained results of the wrist parameters. The people participated in a thorough rehabilitation program to discover the cause of their pronation–supination for 10 days. The workout, which is initiated as soon as the wrist model parameters are retrieved, is under the physiotherapist’s control. For a total of 12 sets, the exercise is performed three times and the rehabilitation robot executes the physiotherapist’s instructions.

The report’s template contains all of the results of the rehabilitation process. The main parameters to be mentioned are the total number of the performed exercises, the movement type of the dedicated joint, the obtained range of motion (degree) in the last session of rehabilitation protocol, the last value of the produced active torque (Nm), and the computed passive torque (Nm) in the last session. On the other hand, three graphs are displayed in the main core of the evaluation report. Indeed, the first one, located at the top right of the report, presents the evolution of the passive torque of the related joint during rehabilitation sessions beginning from the first value detected at the first exercise. The second graph, located at the top left of the report, illustrates the progress of the active torque produced by the related joint while the remaining graph presents the evolution of the joint range of motion with a rising profile. The area at the bottom of the report contains the subject name, the date of the last day of training, and the computed score. Indeed, this score, called the Rehabilitation Progress Factor (RPF), is computed based on the last performed range of motion divided by the maximum allowed range of the motion of a healthy person according to the selected movement type. The following equation explains how to compute RPF based on the obtained range of motion:

$$RPF=\frac{Current\;RoM}{Theoretical\;RoM}*10$$

where *RPF* is the computed score; *Current RoM* is the obtained range of motion of the last rehabilitation session; and *Theoretical RoM* is the allowable joint range of motion for a healthy person.

The people participated in a thorough rehabilitation program to discover the cause of their abduction or adduction for 10 days. The workout, which is initiated as soon as the wrist model parameters are retrieved, is under the physiotherapist’s control. For a total of 12 sets, the exercise is performed 3 times. The linked robot executes the physiotherapist’s instructions. The 3 subjects’ wrists’ RoM had decreased by around 66%, according to the initial tests.

**Figure 15:**
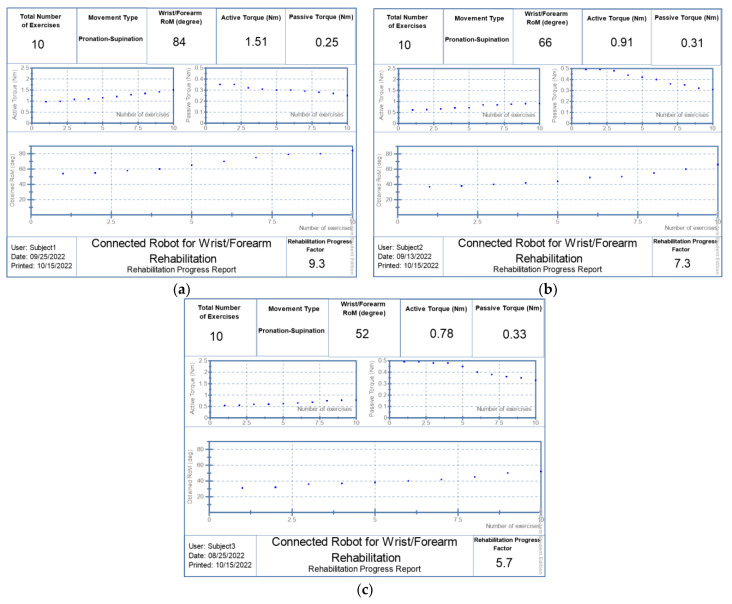
Generated reports: (**a**) patient 1; (**b**) patient 2; (**c**) patient 3.

Subject 1 was received on 14 August 2022. He underwent a 10-day work protocol punctuated by a day of rest. He obtained a RoM of 52 degrees in the pronation–supination movement during the last day (25 August 2022) with RPF equal to 5.7:10, as shown in Figure 15a. Additional rehabilitation activities involved lowering passive stiffness and raising created active torque, in which the subject first reduces the passive torque from 0.49Nm to 0.33Nm. Furthermore, he has a strong capacity to generate additional active torque beginning from 0.51Nm to 0.78Nm achieved in the last day. Likewise, subject 2 was received on 30 August 2022 and he underwent the same protocol. He completed his last session with a RoM of 66 degrees and a RPF of 7.3:10, as shown in Figure 15b, reducing the passive torque to 0.31Nm and producing a 0.91Nm of active torque. Whereas, subject 3 began his rehabilitation protocol on 14 September 2022 and managed to reach a RoM of 84 degrees on 25 September 2022 with an RPF of 9.3:10, as shown in Figure 15c. His passive torque was reduced to 0.25Nm while the provided active torque was increased up to 1.51 Nm.

As shown in Table 6, the’ ROM of the joint of the three subjects was increased by around 66%, compared to the initial state, which corroborates that our robot can be considered as a good tool for a range of motion improvements with very competitive performances compared to similar recent work [45]. On the other hand, the evolution of the estimated joint’s torque shows that the active torque increased by 49% and the passive torque decreased by 30%. This concept is not reported in most recent research that evaluates human joint progress after robotic rehabilitation training [46,47].

Although muscle fatigue estimation during rehabilitation training was already studied by several recent works [48,49,50], the integration of fatigue results in the main controller being challenged. Indeed, in most cases when muscle fatigue is sensed, the defined rehabilitation protocol consists of manually stopping or at least changing the setup of the rehabilitation exercise in order to protect the patient. In this paper, the design of the SVM classifier and the selection of suitable feature extraction approaches are just to prove the high performance of the designed classifier (providing about 90% accuracy). The integration of the classification results into our rehabilitation system is a great challenge.

It is clear that the modeling of human biomechanics and the integration of the developed model into the rehabilitation system is not yet reported in the literature. However, the uncertainty of the human biomechanics model should be quantified to evaluate modeling errors. The adoption of machine learning algorithms for the modeling of the human biomechanics is a good solution for more reliability in the model parameters.

## 4. Conclusions

This article presents a remote-controlled wrist–forearm rehabilitation robot in which the biomechanics of the forearm and wrist joints are integrated into the rehabilitation protocol. The evaluation of the level of rehabilitation state and the extraction of the key elements of the wrist–forearm dynamic model are the two major objectives of wrist modeling. For identifying wrist health status and the dynamic parameters of the human–robot interaction, a new approach based on three steps was proposed. Indeed, in the wrist–forearm robot, the total torque is calculated by adding the torque generated by the robot and any torque provided by the patient, taking fatigue constraints into account. However, patients’ torque is directly affected by fatigue during rehabilitation sessions. To solve this matter, an SVM classifier is integrated into the proposed approach to estimate muscle strain based on features extracted from the electromyogram signal acquired from the patient. Information between the physiotherapist and the patient are communicated through the MQTT protocol. The robot gets the calculated parameters, including the required torque and desired position. Based on the recovered frequential properties from the collected EMG raw, the estimated muscle tiredness is calculated. A human–machine interface was developed to control the rehab robot and provide database space for storing and reporting data. The tests were conducted on three different patients and showed encouraging results.

This work is limited by the reduced number of subjects. In addition, the uncertainty of the human biomechanics model should be evaluated. The next steps consist of moving to the clinical trials by increasing the number of subjects and collecting more reliable results. For example, the integration of machine learning algorithms for the modeling of human biomechanics for more reliability in the model parameters.

## Figures and Tables

**Table 1:** The robot actuators.

Actuators	Weight	Torque
HS-805BB Servomotor	152 g	25 Kg.cm
NEMA 23 stepper motor	500 g	19 Kg.cm

**Table 2:** Movements of the exoskeleton robot.

Joints	Motion	Workspace
Shoulder {1}	Flexion/Extension	0°/140°
Elbow {2}	Flexion/Extension	120°/0°
Forearm {4}	Pronation/Supination	−85°/+85°
Wrist {3}	Ulnar/Radial deviation	−30°/+20°
	Flexion/Extension	+60°/−50°

**Table 3:** Modified Denavit–Hartenberg parameters.

Joint (i)	α_i−1_	a_i−1_	d_i_	*θ*_i_
1	0	0	ds	*θ*_1_
2	0	0	de	*θ*_2_
3	0	0	dw	*θ*_3_
4	π/2	0	0	*θ*_4_

**Table 4:** Definition of the terms in Equation (1).

A	$I_{Hx}\sin^{2}\;\beta +I_{Hy}\cos^{2}\;\beta \;\cos^{2}\;\gamma +I_{Hz}\sin^{2}\;\;\gamma \;\cos^{2}\;\beta +I_{Ay}$
B	$\cos\gamma \cos\beta \sin\gamma \left(I_{Hy}-I_{Hz}\right)$
C	$I_{Hx}\sin\beta$
D	$I_{Hy}\sin^{2}\gamma +I_{Hz}\cos^{2}\;\gamma$
E	0
F	$I_{Hx}$
G	$\begin{array}{ll} I_{Hx}\left[\dot{\beta }\;\cos\beta \left(\dot{\gamma }+2\dot{\alpha }\;\sin\beta \right)\right]\\ & +I_{Hy}[\cos\beta \;\cos\gamma \left(\dot{\beta }\dot{\gamma }\;\cos\gamma -\dot{\alpha }\dot{\beta }\;\sin\beta \cos\gamma -\dot{\alpha }\dot{\gamma }\cos\beta \sin\gamma \right)-\left(\dot{\beta }\;\sin\gamma +\dot{\alpha }\cos\beta \cos\gamma \right)(\dot{\beta }\;\sin\beta \cos\gamma \\ & +\dot{\gamma }\cos\beta \sin\gamma )]\\ & +I_{Hz}[\cos\beta \sin\gamma \left(\dot{\beta }\dot{\gamma }\sin\gamma +\dot{\alpha }\dot{\gamma }\cos\beta \cos\gamma -\dot{\alpha }\dot{\beta }\sin\beta \sin\gamma \;\right)+\left(\dot{\beta }\;\cos\gamma -\dot{\alpha }\cos\beta \sin\gamma \right)(\dot{\beta }\sin\beta \sin\gamma \;\\ & -\dot{\;\gamma }\cos\beta \cos\gamma )] \end{array}$
H	$\begin{array}{ll} -I_{Hx}\dot{\alpha }\;\cos\beta \;\left(\dot{\gamma }+\dot{\alpha }\;\sin\beta \right)\\ & +I_{Hy}[\dot{\alpha }\;\sin\beta \cos\gamma \;\left(\dot{\beta }\sin\gamma +\dot{\alpha }\cos\beta \cos\gamma \right)+\dot{\;\gamma }\cos\gamma \left(2\dot{\beta }\sin\gamma +\dot{\alpha }\cos\beta \cos\gamma \right)\\ & -\dot{\alpha }\sin\gamma \left(\dot{\beta }\sin\beta \cos\gamma +\dot{\;\gamma }\cos\beta \sin\gamma \right)]\\ & +I_{Hz}[\dot{\alpha }\;\sin\beta \sin\gamma \left(\dot{\alpha }\cos\beta \sin\gamma -\dot{\beta }\cos\gamma \right)+\dot{\alpha }\;\cos\gamma \left(\dot{\beta }\sin\beta \sin\gamma -\dot{\;\gamma }\cos\beta \cos\gamma \right)\\ & -\dot{\;\gamma }\sin\gamma \left(2\dot{\beta }\sin\gamma -\dot{\alpha }\cos\beta \sin\gamma \right)] \end{array}$
I	$I_{Hx}\dot{\alpha }\dot{\beta }\cos\beta +\left(I_{Hy}-I_{Hz}\right)\left(\dot{\beta }\sin\gamma +\dot{\alpha }\cos\beta \cos\gamma \right)\left(\dot{\alpha }\cos\beta \sin\gamma -\dot{\beta }\cos\gamma \right)$

**Table 5:** Classification performance indices with the MNF and MNP features.

Frequency Domain Features Extraction	Classifier	Sensitivity (%)	Specificity (%)	Accuracy (%)
MNF	SVM	73.25	85.65	91.32
MNP		82.12	89.45	92.62

**Table 6:** Initial state of the wrist parameters.

Subject Number	Initial Parameters–Obtained Parameters		
	RoM (Degree)	Active Torque (Nm)	Passive Torque (Nm)
**1**	54 to 84	0.97 to 1.51	0.35 to 0.25
**2**	37 to 66	0.61 to 0.91	0.49 to 0.31
**3**	31 to 52	0.54 to 0.78	0.45 to 0.33

## Data Availability

Not applicable.
